# Core fecal microbiota of domesticated herbivorous ruminant, hindgut fermenters, and monogastric animals

**DOI:** 10.1002/mbo3.509

**Published:** 2017-08-22

**Authors:** Michelle M. O’ Donnell, Hugh M. B. Harris, R. Paul Ross, Paul W. O'Toole

**Affiliations:** ^1^ Food Biosciences Department Teagasc Food Research Centre Cork Ireland; ^2^ Department of Microbiology University College Cork Cork Ireland

**Keywords:** diet, herbivore, hindgut, microbiota, monogastric, ruminant

## Abstract

In this pilot study, we determined the core fecal microbiota composition and overall microbiota diversity of domesticated herbivorous animals of three digestion types: hindgut fermenters, ruminants, and monogastrics. The 42 animals representing 10 animal species were housed on a single farm in Ireland and all the large herbivores consumed similar feed, harmonizing two of the environmental factors that influence the microbiota. Similar to other mammals, the fecal microbiota of all these animals was dominated by the *Firmicutes* and *Bacteroidetes* phyla. The fecal microbiota spanning all digestion types comprised 42% of the genera identified. Host phylogeny and, to a lesser extent, digestion type determined the microbiota diversity in these domesticated herbivores. This pilot study forms a platform for future studies into the microbiota of nonbovine and nonequine domesticated herbivorous animals.

## 1 INTRODUCTION

Animal digestion types are classified as herbivores (ruminants and hindgut fermenters), carnivores, and omnivores. Many ruminant microbiota profiling studies have focused on cattle because of their importance in the beef and dairy industry (Brulc et al., 2009; Callaway et al., 2010; Jami & Mizrahi, 2012; Welkie, Stevenson, & Weimer, 2010). Hindgut fermenter microbiota research has similarly focused on the horse because of the economic importance of this species as work and performance animals (Costa et al., 2012; Daly, Stewart, Flint, & Shirazi Beechey, 2001; O’ Donnell et al., 2013; Shepherd, Swecker JR, Jensen, & Ponder, 2012; Steelman, Chowdhary, Dowd, Suchodolski, & Janeäka, 2012). No study to date has used next‐generation sequencing techniques to compare the fecal microbiota of a variety of common domesticated ruminants and hindgut fermenters.

The interplay and symbiotic relationship between the intestinal microbiota and the host are essential for life. Ley, Hamady, et al. (2008), Ley, Lozupone, Hamady, Knight, and Gordon (2008) compared the gut microbiota of over 100 animals to that of humans to assess the composition of the vertebrate microbiota. The study concluded that gut microbiota diversity was influenced by diet type (herbivorous, carnivorous, or omnivorous) and host phylogeny, with herbivorous animals having the most diverse microbiota (Ley, Hamady, et al., 2008). A follow‐up study examined the animal fecal microbiota to assess whether diet or host phylogeny determined the animals microbiota (Muegge et al., 2011). Using Principle Coordinate analysis plots to illustrate the differences between the microbiota, there was a clear separation of carnivores, omnivores, and herbivores. Diet and not phylogeny of the host had the greatest influence on the gut microbiota taxa present (Muegge et al., 2011).

This pilot study aimed to identify the overall fecal microbiota composition and the fecal microbiota of nine species of herbivorous domesticated animal that span two digestion physiologies/types with pigs included as an omnivorous comparator (10 species in total).

## 2 MATERIALS AND METHODS

### 2.1 Animals and diets

All the animals were housed in a mini farm in the south east of Ireland. None of the animals used in the study had received antibiotic treatments in the 12 months prior to sampling. Similarly, none of the animals tested had any health issues prior to sampling and are thus considered to be healthy animals. A list of each animal (and the sample number of each) and the feed consumed by each are given in Table 1. The Kingdom, Phylum, Class, Order, and Family for each animal species are listed in Table S1. Twenty‐five hindgut fermenting, 16 ruminant, and 4 monogastric animals were used in this study, spanning 10 animal species. Animals that were housed indoors (rabbits, chinchillas, and pigs) were fed twice daily and had access to water *ad libitum*. The other animals were kept on pasture paddocks and therefore fed naturally by grazing also with access to water *ad libitum*. Animals of the same species were frequently cohoused indoors or together in large, open paddocks. This reflects a more natural, active farm environment.

**Table 1:** Animals, diets, and 16S gene amplicon sequence reads generated to study their gut microbiota

Animal	Binomial nomenclature	Abbrev.	*n*	Digestion type	Feed supplied	Sequence reads
Chinchilla	*Chinchilla lanigera*	Ch	3	Hindgut fermenter	Commercial feed^a^	37,013
Rabbit	*Oryctolagus cuniculus*	Ra	8	Hindgut fermenter	Commercial feed^b^	74,963
Donkey	*Equus africanus asinus*	Do	7	Hindgut fermenter	Grass	220,774
Miniature pony	*Equus ferus caballus*	MP	7	Hindgut fermenter	Grass	46,884
Deer	*Cervus nippon*	De	4	Ruminant	Grass	32,635
Goat	*Capra aegagrus hircus*	Go	5	Ruminant	Grass	27,791
Sheep	*Ovis aries*	Sh	4	Ruminant	Grass	43,559
Llama	*Lama glama*	Ll	2	Ruminant	Grass	52,461
Alpaca	*Vicugna pacos*	Al	1	Ruminant	Grass	10,837
Pig	*Sus scrofa scrofa kunekune*	Pi	2	Monogastric	Sow pellets and bread	14,040

a Dehydrated grass pellets, alfalfa pellets, chopped alfalfa hay, flaked field peas, flaked corn, vitamins, and minerals.

b Dry grass flaked maize, carrots, corn, and oat grains supplemented with additional carrots.

### 2.2 Fecal sample collection, DNA extraction, and 454 pyrosequencing

Fresh fecal samples were collected from each animal, placed in sterile 100 ml pots and frozen at −80°C. Total bacterial genomic DNA was isolated from the feces according to the Repeat Bead Beating plus column method (RBB+C) (Yu & Morrison, 2004). The extracted DNA was then used as a template in the V4 region PCR amplifications using a method outlined previously (O’ Donnell et al., 2013). Samples were sequenced with 454 Titanium technologies (Teagasc Food Research Centre, Moorepark, Ireland).

### 2.3 Sequence processing and OTU clustering

Raw 16S V4 reads were processed and analyzed using Qiime 1.5.0. Reads with any of the following criteria were removed from the dataset: shorter than 150 bp; longer than 350 bp; one or more errors in the barcode; two or more errors in the primer; a quality score that dropped below an average of 25 (phred) in a sliding window of size 50 bp. Upon demultiplexing, barcodes and primers were removed. All reads that passed quality filtering were clustered into OTUs at 97% identity using UCLUST (Edgar, 2010). For each OTU, a representative sequence was chosen using the Qiime default (most abundant sequence at 100% identity). Representative sequences were aligned using PyNAST (Caporaso et al. 2009) using the best match from the GreenGenes core set (Desantis et al., 2006). Taxonomy was assigned from phylum to genus level using the RDP classifier (Cole et al. 2005) with a minimum confidence value of 0.5. Chimeric sequences were removed using ChimeraSlayer (Haas et al., 2011). Singleton OTUs were removed where a singleton stands for a single read present in a single sample. A phylogenetic tree was built from the aligned representative set using FastTree (Price, Dehal, & Arkin, 2009). The OTU table was rarefied to account for variations in sequencing depth among the samples. Weighted and unweighted UniFrac (Lozupone & Knight, 2005) distances were computed from the rarefied OTU table and these were used to generate PCoA plots in R 2.15.1. To define a core taxa, the following criteria were used (a) present at ≥0.1% of total reads and (b) present in >2 digestion types or 5 animal species. The median read proportions at each taxon level for each species were pooled to form the species datasets. The median proportions of each species were then pooled to generate the three digestion type datasets. VENNY, an online Venn diagram tool was used to create a figure representing the core genera (Oliveros, 2007). A heatmap for the genera present in each animal species was generated using R (Team, R. C, 2014) and Bioconducter (Gentleman et al., 2004). A genus whose relative read abundance is less than 1% (of the total) in at least one sample was removed. The Bray–Curtis dissimilarity matrix with average linkage hierarchical clustering was used to cluster the species together. The same matrix was used to cluster genera that occurred together more frequently.

### 2.4 Alpha and beta diversity matrices

Five alpha diversity metrics were calculated to measure the microbial diversity in the three digestion types and in each animal species. These metrics were Observed Species (OTU count), Phylogenetic Diversity, the Shannon index, Simpson's index, and Good's coverage. Each metric was calculated from a rarefied OTU table consisting of subsamples of 2,440 reads per sample. Observed Species, Shannon index, and Phylogenetic Diversity metrics were calculated as previously outlined (O’ Donnell et al., 2013). Simpson's index (D) measures the probability that two individuals randomly selected from a sample will belong to different OTUs. Good's coverage or Estimated Sample Coverage (ESC) was estimated using the formula ESC = 1 – n/N, where n = number of singleton OTUs and N = number of assigned reads. A second subset of 10,000 reads was also used to generate rarefaction curves, to plot the alpha diversity in the hindgut fermenters (*n* = 8) and ruminants (*n* = 6). Each animal chosen as a representative of its digestion type had read assignments greater than 10,000 reads.

Beta diversity Principle Coordinate of Analysis (PCoA) plots were calculated as previously described (O’ Donnell et al., 2013).

### 2.5 Statistics

The Mann–Whitney test (Siegel, 1956) was used for all pairwise comparisons in this study and, in cases where multiple correction of *p*‐values were necessary, Benjamini & Hochberg (1995) was used. Before statistics were carried out on the data, each group of taxa from phylum to species was filtered for those that were present in 50% of samples or greater; this ensured that the number of zero values was not heavily biased in one group over the other, which would lead to inaccurate *p*‐values. Statistics were only performed on groups where the sample size was >=4; this was true for comparison of the hindgut fermenters and ruminants (monogastric animals were omitted for low sample size) and also for comparison of the 10 animal groups.

The Adonis function in R package was applied to the weighted and unweighted UniFrac distances for the animal groups (*n* = 10) and digestion groups (*n* = 3).

### 2.6 Data Availability

Meta data file for processing sequences in Qiime: https://doi.org/10.6084/m9.figshare.4970174. Forward sequence reads: https://doi.org/10.6084/m9.figshare.4970138. Reverse sequence reads: https://doi.org/10.6084/m9.figshare.4970153. Forward quality files for reads: https://doi.org/10.6084/m9.figshare.4970159. Reverse quality files for reads: https://doi.org/10.6084/m9.figshare.4970162.

## 3 RESULTS

We used 16S rRNA gene (V4 region) amplicon pyrosequencing to determine the fecal microbiota composition of 10 animal species totaling 42 individual animals (having removed two of the porcine datasets because of low read counts). The total number of reads identified following filtering and chimera identification was 560,957, with read numbers per animal/fecal sample ranging from 2,440 to 100,544 (Table 1). The average read length was 207 bp. Assignments to the Bacterial kingdom accounted for a median 96% of the total reads in each animal with a median 0.01% of the reads assigned to the Archaea. At each taxon level, only the microbiota of *Equidae* (donkeys and miniature ponies) and sheep contained members of the *Archaea*, specifically *Methanocorpusculum* and *Methanobrevibacter*. The remaining phylum level reads were uncharacterized read assignments (between 3% and 4% for the three digestion types).

### 3.1 Dominant taxa in the fecal microbiota

The predominant phyla identified in the three digestion types and across the 10 animal species were *Firmicutes* and *Bacteroidetes*. The abundance of the *Firmicutes* phylum was significantly higher (*p* ≤ .05) in the ruminants compared to the hindgut fermenters. The dominant phylogenetic assignments for each digestion type are listed in Table 2 **.** The dominance of the *Firmicutes* phylum in the microbiota of domesticated herbivores was reflected in the other predominant taxa identified (*Clostridia* > *Clostridiales* > *Ruminococcaceae* > *Sporobacter*). *Actinobacteria* was identified as a dominant phylum in the microbiota of rabbits. The predominance of this phylum in the rabbit microbiota was reflected throughout the lower level taxonomic data (*Actinobacteria* > *Bifidobacteriales* > *Bifidobacteriaceae* > *Bifidobacterium*). The dominance of *Betaproteobacteria* in the chinchilla microbiota was the sole host animal‐specific class identified in this study. Host animal‐specific dominant orders included *Burkholderiales* (chinchillas) and *Verrucomicrobiales* (rabbits and sheep). Host animal‐specific families identified included *Marinilabiaceae* (donkeys and miniature ponies), *Chitinophagaceae* (deer), and *Moraxellaceae* (llamas). The predominant genus in the fecal microbiota of the monogastric animal was *Treponema*. Host animal‐associated dominant genera were identified in the chinchillas (*Parabacteroides* and *Barnesiella*), rabbits (*Persicirhabdus* and *Subdoligranulum*), donkeys (*Anaerophaga*), llamas (*Hydrogenoanaerobacterium* and *Acinetobacter*), and alpacas (*Roseburia*). *Galbibacter* and *Clostridium* were identified as dominant genera in the equids and camelids, respectively. Statistically significant differences observed between the abundances of particular microbiota elements between ruminants and hindgut fermenters are presented in Table S2.

**Table 2:** Dominant taxa in the microbiota associated with three digestion types (percentage proportional abundance)

Taxon	Digestion type		
	Hindgut	Ruminant	Monogastric
Phylum			
Firmicutes	53.11	65.35	52.27
Bacteroidetes	31.36	20.95	26.95
Verrucomicrobia	2.90	1.24	0.54
Spirochaetes	1.93	0.91	10.34
Proteobacteria	1.68	1.52	3.44
Class			
Bacteroidia	8.26	10.67	7.37
Flavobacteria	4.60	0.75	2.26
Sphingobacteria	2.15	4.96	3.33
Bacilli	0.37	0.12	1.08
Clostridia	45.91	62.65	48.83
Erysipelotrichia	1.17	0.86	1.38
Alphaproteobacteria	0.23	0.45	0.12
Deltaproteobacteria	0.18	0.37	0.47
Spirochaetes	1.93	0.91	10.34
Subdivision5	1.07	0.10	0.31
Order			
Bacteroidales	8.26	10.67	7.37
Flavobacteriales	4.60	0.75	2.26
Sphingobacteriales	2.15	4.96	3.33
Clostridiales	44.09	60.73	48.31
Erysipelotrichales	1.17	0.86	1.38
Spirochaetales	1.93	0.91	10.34
Subdivision5	1.07	0.10	0.31
Family			
*Bacteroidaceae*	0.36	1.85	0.32
*Porphyromonadaceae*	2.10	3.73	3.06
*Prevotellaceae*	2.09	1.41	2.93
*Flavobacteriaceae*	3.40	0.64	1.69
*Sphingobacteriaceae*	1.97	0.55	2.44
*Clostridiaceae*	0.27	0.44	0.43
Clostridiales Family XIV. Incertae Sedis	0.50	0.20	0.78
*Eubacteriaceae*	0.28	0.23	0.65
*Lachnospiraceae*	6.84	5.26	3.30
*Ruminococcaceae*	20.48	33.46	23.97
*Erysipelotrichaceae*	1.17	0.86	1.38
*Veillonellaceae*	0.82	0.76	2.88
*Spirochaetaceae*	1.87	0.82	10.34
Genus			
*Bacteroides*	0.36	1.85	0.32
*Prevotella*	0.91	0.36	2.38
*Anaerosporobacter*	0.15	0.11	0.11
*Clostridium*	0.16	0.28	0.33
*Butyricicoccus*	0.13	0.24	0.80
*Eubacterium*	0.18	0.19	0.63
*Blautia*	0.50	0.2	0.78
*Coprococcus*	0.42	0.89	0.82
*Oscillibacter*	0.71	1.55	1.74
*Hydrogenoanaerobacterium*	0.18	0.34	0.31
*Anaerotruncus*	0.35	0.37	0.46
*Acetivibrio*	0.93	1.25	0.60
*Papillibacter*	0.45	1.65	0.93
*Faecalibacterium*	1.10	0.34	2.92
*Ruminococcus*	2.29	1.78	2.98
*Sporobacter*	3.63	5.05	4.34
*Acidaminococcus*	0.33	0.10	0.30
*Treponema*	1.87	0.82	10.33

### 3.2 Core microbiota of domesticated herbivores

To define a core taxa, the following criteria were used (a) present at ≥0.1% of total reads and (b) present in >2 digestion types or 5 animal species. Firmicutes, Bacteroidetes, Verrucomicrobia, Spirochaetes, and Proteobacteria were identified as the core phyla in the fecal microbiota of the domesticated herbivores (Table 2). These five phyla were also noted as the dominant phyla in each animal species. Eighteen core genera were identified as being shared across the three digestion types (Figure S1). *Acidaminobacter*,* Anaerophaga*,* Dorea*,* Fibrobacter*,* Lactobacillus*,* Subdoligranulum*, and *Parabacteroides* were identified as core hindgut fermenter‐associated genera. *Acetanaerobacterium*,* Acetitomaculum*,* Croceibacter*,* Holdemania*,* Lutispora, Persicirhabdus,* and *Victivallis* were identified as core ruminant microbiota‐associated genera. Monogastric microbiota‐associated core genera identified in this study were *Bulleidia*,* Catenibacterium*,* Hespellia*,* Lysinibacillus*,* Megasphaera*,* Parasporobacterium*,* Petrimonas,* and *Pseudomonas*. *Akkermansia*,* Alistipes*,* Paludibacter*,* Paraprevotella*,* Robinsoniella,* and *Roseburia* were recognized as the six additional genera forming the core microbiota shared by the hindgut fermenters and ruminants.

Forty‐two percent of the genera (42/100 genera) were identified as forming the core microbiota across the digestion types. Thirty‐three genera were identified as forming the core microbiota of domesticated herbivores across the animal species and are presented in Table 3. The majority of these genera were members of the *Clostridiales* order.

**Table 3:** The core fecal microbiota at genus level of the animals studied (percentage proportional abundance)

Genus	Animal									
	Chinchilla	Rabbit	Donkey	Miniature pony	Deer	Goat	Sheep	Llama	Alpaca	Pig
*Bacteroides* a	3.38	2.29	0.11	0.16	2.39	3.14	2.0	1.37	0.92	0.32
*Paludibacter* a	0.00	0.00	0.96	0.68	3.44	1.53	0.27	1.36	1.45	0.06
*Parabacteroides*	2.37	0.49	0.24	0.00	0.06	0.11	0.10	0.19	0.27	0.10
*Paraprevotella*	0.37	0.09	0.23	2.34	0.14	0.63	0.38	0.64	0.96	0.04
*Prevotella* a	1.28	0.53	0.89	1.49	0.10	1.42	0.44	0.54	0.39	2.38
*Alistipes*	0.52	0.4	0.07	0.02	4.51	5.43	2.24	0.25	0.06	0.02
*Galbibacter*	0.00	0.00	3.51	5.56	0.00	0.02	0.14	0.6	0.83	1.02
*Anaerosporobacter* a	0.04	0.10	0.17	0.23	0.05	0.10	0.11	0.13	0.35	0.11
*Butyricicoccus* a	0.29	0.22	0.07	0.12	0.18	0.29	0.18	0.28	0.21	0.80
*Clostridium* a	0.00	0.01	0.38	0.16	0.32	0.16	0.20	1.8	1.67	0.33
*Lactonifactor*	0.02	0.00	0.14	0.29	0.04	0.30	0.51	0.12	0.07	0.01
*Lutispora*	0.00	0.00	0.00	0.01	0.36	0.31	0.32	0.01	0.04	0.01
*Acidaminobacter*	0.05	0.22	0.18	0.10	0.36	0.00	0.17	0.51	0.00	0.05
*Eubacterium* a	0.19	0.62	0.06	0.12	0.43	0.22	0.14	0.12	0.27	0.63
*Blautia* a	0.61	0.99	0.26	0.43	0.13	0.22	0.20	0.38	0.84	0.78
*Coprococcus* a	0.58	0.35	0.41	0.44	0.95	0.96	0.80	0.97	1.05	0.82
*Dorea*	0.24	0.42	0.04	0.08	0.12	0.11	0.07	0.09	0.29	0.01
*Oribacterium*	0.05	0.03	0.05	0.18	0.00	0.12	0.10	0.26	0.23	0.04
*Robinsoniella*	0.43	0.01	0.29	0.23	0.35	0.30	0.15	0.16	0.08	0.09
*Roseburia*	0.27	0.3	0.25	0.89	0.33	0.26	0.14	0.14	1.03	0.00
*Oscillibacter* a	0.58	0.3	1.38	0.98	1.30	1.74	1.95	1.57	1.42	1.74
*Acetivibrio* a	0.89	0.32	1.05	0.99	1.17	1.06	1.70	1.65	1.37	0.60
*Anaerotruncus* a	0.17	0.23	0.61	0.47	0.52	0.39	0.51	0.16	0.19	0.46
*Faecalibacterium* a	1.21	2.77	0.50	1.04	0.21	0.80	0.56	0.42	0.51	2.92
*Hydrogenoanaerobacterium* a	0.75	0.09	0.18	0.26	0.59	0.27	0.18	1.24	0.48	0.31
*Papillibacter*	1.1	0.22	0.65	0.52	1.60	2.11	2.32	1.27	1.14	0.93
*Ruminococcus* a	5.65	14.23	1.17	1.67	1.63	1.64	1.79	2.34	1.61	2.98
*Sporobacter* a	0.63	4.29	3.42	5.02	5.15	5.09	4.67	4.47	2.88	4.34
*Holdemania*	0.01	0.02	0.04	0.13	0.16	0.29	0.10	0.20	0.11	0.01
*Acidaminococcus* a	0.19	0.1	0.36	0.42	0.10	0.11	0.08	0.24	0.55	0.30
*Treponema* a	0.03	0.14	6.55	2.02	1.24	0.85	0.52	2.78	6.51	10.33
*Akkermansia*	0.00	0.25	0.83	0.02	0.48	0.37	0.28	0.41	0.04	0.00
*Persicirhabdus*	0.00	2.96	0.03	0.00	0.21	0.18	0.77	0.14	0.00	0.00

a The 18 core genera identified from the three digestion types.

**Figure 1:**
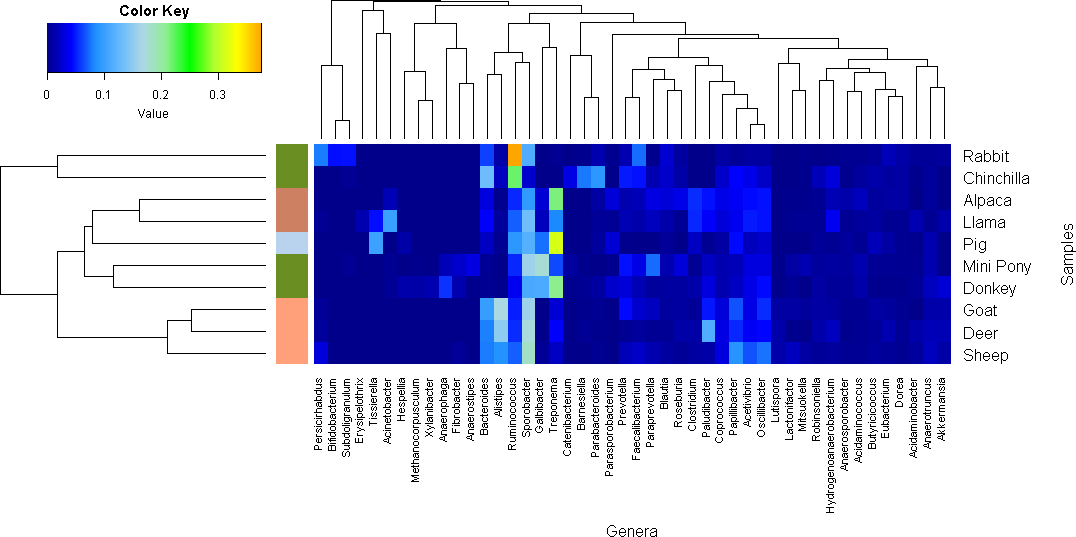
Heatmap of the median percentage relative abundance of any genus above 1% in the 10 different animal species. Animal digestion types are labeled on the *y*‐axis of the plot according to the following colors; Hindgut fermenters = olive, Ruminants = Salmon, Pseudoruminants = Light salmon, Monogastric = Grey

By comparing the genera from the animal species using a Bray–Curtis dissimilarity matrix heatmap, two major animal clusters were identified (Figure 1); one cluster contained the rabbits and chinchillas (smaller hindgut fermenters) and the other cluster contained the remaining larger animals. The larger animal cluster, containing the majority of the animals, is separated into two further minor clusters. These clusters separate the ruminants from the large hindgut fermenters, monogastric fermenters, and the pseudoruminants. The genera then were clustered into seven different clusters. Of particular note was one of the clusters which contained the *Bacteroides*,* Alistipes*,* Ruminococcus*,* Sporobacter*,* Galbibacter,* and *Treponema* genera. This cluster appears to be a major distinction between the ruminants, pseudoruminants, and larger hindgut fermenters. Pseudoruminants have a three‐chambered stomach instead of four, like ruminants and include alpacas and llamas.

### 3.3 Microbiota diversity differences between digestion types and animals

The alpha diversity of the ruminant fecal microbiota was greater than that of the hindgut fermenters, as measured with the Shannon diversity index and OTU counts (*p* < .01 and *p* < .05, respectively) indices. Rarefaction curves were generated from 2,440‐read subsets of the populations (Figure S2). The phylogenetic diversity and OTU count curves failed to reach a saturation plateau for any of the digestion types/animals, indicating that the sampling depth in this study failed to capture the complete microbiota diversity. However, both the Shannon diversity and Simpson diversity index plots plateaued, suggesting that further sampling would not yield additional phylotypes. The Good's coverage metric was used to estimate the completeness of sampling, with median coverage percentages of 90% to 96%. The Good's coverage percentages for each sample also indicate that, like the Shannon and Simpson diversity indices, further microbiota sampling would result in a small number of additional phylotypes.

Table 4 summarizes the alpha diversity indices for the individual animal species. The donkey microbiota was the most diverse, while the rabbit fecal microbiota was the least diverse. This difference between animals with a similar digestion type may be due to the relative size of the animals and the longer gut retention times of the equids.

**Table 4:** Alpha diversity metrics in the digestion types studied

Digestion type	Animal species	Diversity metric (SD)					
		Phylogenetic Diversity	Shannon Weaver	Simpson index	Chao1 score	Observed species	Good's coverage (ESC)
Hindgut fermenters	Chinchillas	38.97 (13.45)	6.78 (1.92)	0.973 (0.10)	1027 (619)	445 (225)	95% (4.01)
	Rabbits	41.03 (7.92)	6.43 (0.72)	0.962 (0.02)	865 (341)	415 (104)	95% (4.26)
	Donkeys	62.66 (6.02)	7.82 (0.22)	0.988 (0.00)	1278 (164)	606 (64)	92% (5.08)
	Miniature ponies	49.08 (6.84)	7.37 (1.41)	0.987 (0.13)	910 (191)	472 (121)	91% (2.88)
Ruminants	Deer	52.01 (1.00)	7.69 (0.17)	0.979 (0.00)	1112 (81)	614 (26)	90% (2.38)
	Goats	57.78 (6.54)	8.00 (0.35)	0.987 (0.00)	1262 (129)	660 (73)	87% (3.48)
	Sheep	58.22 (1.68)	7.95 (0.15)	0.986 (0.00)	1144 (36)	645 (22)	92% (2.82)
	Llamas	54.78 11.61)	7.43 (0.61)	0.982 (0.01)	1028 (369)	542 (171)	95% (4.42)
	Alpaca	48.87 (N/A)	7.22 (N/A)	0.979 (N/A)	844 (N/A)	472 (N/A)	97% (N/A)
Monogastric	Pigs	48.22 (3.7)	6.98 (0.04)	0.976 (0.00)	739 (8)	434 (27)	94% (0.74)

N/A – single animal therefore we were unable to calculate standard deviation.

### 3.4 Clustering of the intestinal microbiota by digestion type and host phylogeny

**Figure 2:**
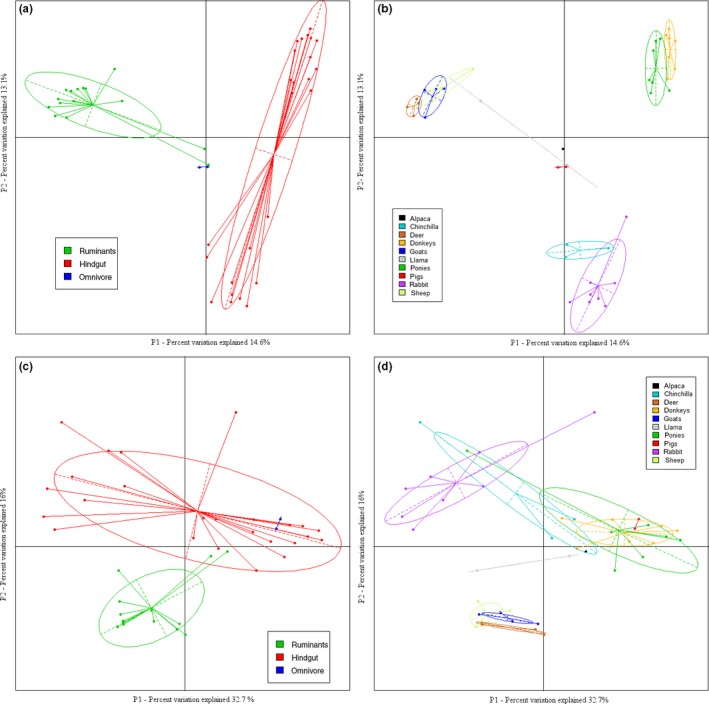
UniFrac beta diversity measures (a) unweighted plot for the microbiota of three digestion types (b) unweighted plot for the microbiota of the 10 animal species (c) weighted plot for the microbiota of the three digestion types (d) weighted plot for the microbiota of the 10 animal species

Principal coordinate analysis (PCoA) plots constructed by UniFrac with unweighted and weighted taxon abundance values were used to visualize and examine the beta diversity of both the digestion types and the animal species (Figure 2). The low variance explained by the first two axes (27.7%) in the unweighted plots is common when many diverse factors may affect the samples. The first two axes in the weighted plots accounted for 48.7% of the variance. The unweighted and weighted PCoA plots showed a clustering of bacteria within each microbiota by the digestion type (Figure 2a and c). However, there was an overlap between the microbiota from the monogastric animal species (pig) and those from the hindgut fermenters in the weighted PCoA plot (Figure 2c). The output of our Adonis analysis using UniFrac distance matrix was significant (*p* < .001). The weighted PCoA animal species microbiota plots (Figure 2d) showed a clustering of the microbiota of each animal species based on their Family as well as digestion type. Groupings include the *equidae* (donkeys and miniature ponies; hindgut fermenters), *camelidae* (llama and alpaca; ruminants/pseudoruminants), and *bovidae* (sheep and goats; ruminants). The remaining animal species’ microbiota appear to cluster based on the digestion type and Order (*Artiodactyla*). This suggests that host phylogeny, which in our case, is determined by digestion type may largely determine the microbiota of the herbivorous domesticated animals studied. However, it should be noted that digestion type and host phylogeny are not independent of each other and closely related animal species are more likely to share the same digestion type (e.g., goats and sheep).

## 4 DISCUSSION

The objective of this pilot study was to identify the bacterial taxa present in hindgut fermenters and ruminant animals dwelling on a single farm. Recently, the fecal microbiota of both rabbits (Eshar & Weese, 2014) and donkeys (Liu et al., 2014) was investigated by researchers from the US and China, respectively. A similar study carried out focusing on ruminants only identified a core microbiome spanning a wide geographic area (Henderson et al., 2016). This study is the first to report and investigate the microbiota of rabbits and donkeys residing in Ireland. The colocalization of the large herbivores in particular, studied here removes the geographic, management regime, and diet differences noted in other studies (O’ Donnell et al., 2013; Shanks et al., 2011; Yamano, Koike, Kobayashi, & Hata, 2008). In this study, we showed that the domesticated herbivorous animals shared a common fecal microbiota but that some genera were associated with particular digestion types only. Inherent differences exist between the microbiota from different sampling sites within the herbivore gut (Dougal, de la Fuente, et al., 2013). The reliance on fecal samples in this study is not without issues/concerns, however, the high bacterial numbers (10^14^) within the colon of both humans and animals gives credence to the use of fecal material in studies. Fecal sampling serves as an alternate for more laborious and invasive sampling from, in these circumstances, commercial, domesticated livestock from an active farm.

The phyla Firmicutes and Bacteroidetes were identified as the predominant phyla in the microbiota of all the domesticated herbivores in this study; however, this trend has not been universal for other studies and animals (Barker, Gillett, Polkinghorne, & Timms, 2013; García‐Amado et al., 2012; Ishaq & Wright, 2012; Li et al., 2013). Ley et al. identified Firmicutes and Bacteroidetes as the phyla found most ubiquitous in the vertebrate microbiota (Ley, Hamady, et al., 2008; Ley, Lozupone, et al., 2008). Although controversial/contentious (Schwiertz et al., 2010), the Firmicutes: Bacteroidetes ratio has also been implicated as factor in the health status of vertebrates (Ley et al., 2005). These phyla accounted for 79%–86% of the total microbiota in the domesticated herbivores. The Firmicutes: Bacteroidetes ratio for the hindgut fermenters and monogastric fermenter was <2. This is lower than the ratio of 3:1 noted for the ruminant animals in this study and also noted by Ley, Lozupone, et al., (2008) for other vertebrates. This suggests that Bacteroidetes play a greater role in the hindgut and monogastric fermenter microbiota than in ruminants. This correlates with a statistically higher proportion of reads assigned to the Firmicutes phylum in the ruminant microbiota.

In recent years, more focus and attention has been paid to the microbiota of domesticated hindgut fermenters, in particular, horses (Barker et al., 2013; Costa et al., 2012; O’ Donnell et al., 2013; Shepherd et al., 2012; Steelman et al., 2012). The core fecal microbiota families of large hindgut fermenters have been identified and include *Erysipelotrichaceae, Ruminococcaceae*,* Lachnospiraceae*,* Prevotellaceae,* and *Rikenellaceae* (Bian, Ma, Su, & Zhu, 2013; Dougal, de la Fuente, et al., 2013a). The same families were also identified as core families in the hindgut fermenters studied. In this study, we also identified *Spirochaetaceae*,* Porphyromonadaceae*,* Flavobacteriaceae*,* Bacteroidaceae,* and *Clostridiaceae* as core families in the domesticated herbivores studied. The proportion of the *Bacteroidetes* phylum was higher in the microbiota of the miniature ponies study than in the grass fed horses we previously studied (O’ Donnell et al., 2013) but lower than in the fecal microbiota of other horses (Dougal, de la Fuente, et al., 2013). The difference may be due to the effect that different DNA extraction methods can have on the data generated (Henderson et al., 2013). We previously identified *Ruminococcus*,* Sporobacter,* and *Treponema* as dominant genera in the equine hindgut (O’ Donnell et al., 2013), genera that were also identified in this study as dominant in the hindgut fermenters (chinchillas, rabbits, miniature ponies, and donkeys). *Fibrobacter* was also identified as an important genus particularly for the hindgut‐fermenting equids; this is consistent with previous studies (Shepherd et al., 2012).

The reasons for the differences in the dominant phyla between the studies and animals may be multi‐factorial and include the different diets consumed, geographic locations, PCR amplification bias or, as noted above, due to the DNA extraction methods employed (Berry, Ben Mahfoudh, Wagner, & Loy, 2011; de Carcer et al., 2011; De Filippo et al., 2010; Henderson et al., 2013). The use of “mock” bacterial communities within studies can also aid in controlling bias (Ahn, Kim, Song, & Weon, 2012). The ruminant digestive tract and its microbiota have evolved to degrade the fibrous plant material consumed (Clauss, Hume, & Hummel, 2010; Mackie, 1997). The majority of the core ruminant microbiota‐associated genera identified in the study have been previously identified in the other ruminants at varying proportions (Callaway et al., 2010; Greening & Leedle, 1989; Reti, Thomas, Yanke, Selinger, & Inglis, 2013). Many genera have been identified as rumen‐associated bacteria involved primarily in, but not restricted to, the digestion of plant polysaccharides. Important plant polysaccharide‐associated degrading bacteria include *Ruminococcus*,* Prevotella*,* Butyrivibrio,* and *Alistipes* (Dowd et al., 2008; Kim, Morrison, & Yu, 2011) all of which were identified in this study. However, only the *Succiniclasticum* and *Butyrivibrio* genera were associated with the microbiota of ruminants alone, and at very low proportions (<0.2%). Additional genera potentially involved in plant polysaccharide utilization have been identified in both marine and terrestrial herbivores including *Anaerotruncus*,* Roseburia*,* Oscillibacter*,* Bacteroides*,* Coprococcus,* and *Blautia* (Nelson, Rogers, & Brown, 2013; Yildirim et al., 2010). All of these taxa were identified at >0.1% of the total reads in the hindgut fermenter, ruminants, and monogastric animals studied.

Studies of other domesticated ruminants have identified the potential effect of the different diets on the genera identified (De Jesús‐Laboy et al., 2012; Li et al., 2013). *Prevotella* was previously identified as the dominant genus in the sika deer rumen microbiota (Li et al., 2013); however, in this study, *Sporobacter* was the dominant genus identified in the fecal microbiota of the sika deer. While examining the effects that domestication can have on an animal species microbiota, De Jesús‐Laboy et al. (2012) noted that the Actinobacteria phylum was present in all the domesticated goats studied. We failed to detect the Actinobacteria phylum in the domesticated pygmy goat microbiota. In contrast, the Actinobacteria phylum was associated with the hindgut‐fermenting animals and, in particular, the rabbits. Geographical distance/location may explain the differences in the predominant genera identified (Dougal, Harris, et al., 2013; Pei et al., 2010).

The Kune‐kune pigs used in this study, while classed as monogastric fermenters (omnivores), are considered to be primarily herbivorous. This overlap of an omnivorous animal microbiota with that of the hindgut fermenters in the weighted PCoA beta diversity plot has been observed in other studies (Nelson et al., 2013). The weighted PCoA plots (which include proportional data) displayed the animal species microbiota clustered by their families and digestion type, with the true ruminant animals (deer, goats, and sheep) clustered by digestion type and Order. Ley, Hamady, et al. (2008) identified the herbivorous microbiota as the most diverse when compared to omnivores and carnivores. Our study expanded on this by focusing only on herbivorous animals and within these parameters, we noted that the ruminant fecal microbiota is more diverse than the hindgut microbiota. A caveat to the use of the Kune‐kune pigs in this study is the interpretation of host‐associated taxa from a small sample size. Future studies will need to expand upon the knowledge of the monogastric fecal microbiota using larger numbers of pigs.

The proportion of unclassified reads identified at the genus level in this study is consistent with other studies carried out on humans, hindgut fermenters, and less commonly studied ruminants (Claesson et al., 2009; Janssen & Kirs, 2008; O’ Donnell et al., 2013). The high percentage of unclassified read proportions is due to the short amplicon sequence read length used, compounded by the lack of culturing and sequence identification work on the more obscure hindgut fermenters and ruminants (Pei et al., 2010). The high percentage of unclassified reads at genus level is due to the short read length of the 16S V4 region and the limited size and diversity of the RDP database. As sequencing projects proceed at an increasing pace, the diversity of microbial sequences belonging to the microbiota of hindgut fermenters and ruminants in databases such as RDP is likely to grow. Accompanied by improvements in sequencing technologies that allow for longer reads, these growing databases will lead to a more comprehensive classification of 16S reads at the genus level.

The diversity indices indicated that while the ruminant microbiota was more diverse than the hindgut‐fermenting counterparts, compared to other microbiota sequencing studies, they are less diverse. We measured lower phylotype diversity in the hindgut fermenter, ruminant, and monogastric microbiota compared to data from the distal bowel microbiota of other animals (Lamendella, Santo Domingo, Ghosh, Martinson, & Oerther, 2011; Pitta et al., 2010). Our phylotype estimations for the animal species (415–660) were within the ranges estimated for the human microbiota (Claesson et al., 2009; Nam, Jung, Roh, Kim, & Bae, 2011) but lower than our previous hindgut microbiota estimates (O’ Donnell et al., 2013). The failure of the OTU count and phylogenetic diversity rarefaction curves to plateau indicated that complete sampling of the domesticated herbivore fecal microbiota has not been achieved, despite sequencing over 10,000 reads per fecal sample. Good's coverage ranged from 90% to 96% for the animal species, indicating that 8–33 additional reads would need to be sequenced to detect a new phylotype (Claesson et al., 2009). This level of coverage indicates that the 16S rDNA V4 sequences identified in these samples represent the majority of bacterial sequences present in the domesticated herbivore microbiota. The Good's coverage estimates are consistent with those for humans, hindgut‐fermenting mammals and larger than for some ruminants (Berry et al., 2011; Janssen & Kirs, 2008; Nam et al., 2011). However, there are caveats to bear in mind when comparing and interpreting the differences in the diversity present in a particular microbiota or study. Each study may be affected by the method used to generate the data and assignments (Kemp & Aller, 2004).

In conclusion, in this pilot study, we have shown that the hindgut fermenting, ruminant, and monogastric microbiota share 50% of their phyla and over 15% of their genera in their fecal microbiota. This degree of overlap between the microbiota of the 10 animal species may suggest that these genera are essential for all herbivorous fibrous polysaccharide‐consuming animals. Host phylogeny and digestion method were shown to be potential determinants of bacterial diversity in the domesticated herbivores. Further studies in larger multi‐animal farms in other countries would help to confirm our findings and identify other determinants shaping the diversity in the animal microbiota. Longitudinal studies of colocalized animals would also facilitate the examination of the effect that seasonal variation (Hoffman et al., 2001; Kobayashi, Koike, Miyaji, Hata, & Tanaka, 2006; Mathiesen, Orpin, Greenwood, & Blix, 1987) in feed consumed could have on the microbiota of domesticated herbivores.

## CONFLICT OF INTEREST

No conflicts of interest declared.
