# Supplementary material for: Core fecal microbiota of domesticated herbivorous ruminant, hindgut fermenters, and monogastric animals
Source: Microbiologyopen. 2017 Aug 22;6(5):e00509. doi: 10.1002/mbo3.509 (PMC5635170; doi:10.1002/mbo3.509)
Supplement: Supplementary file 1 [file MBO3-6-na-s001.docx]

# Supplementary information

**
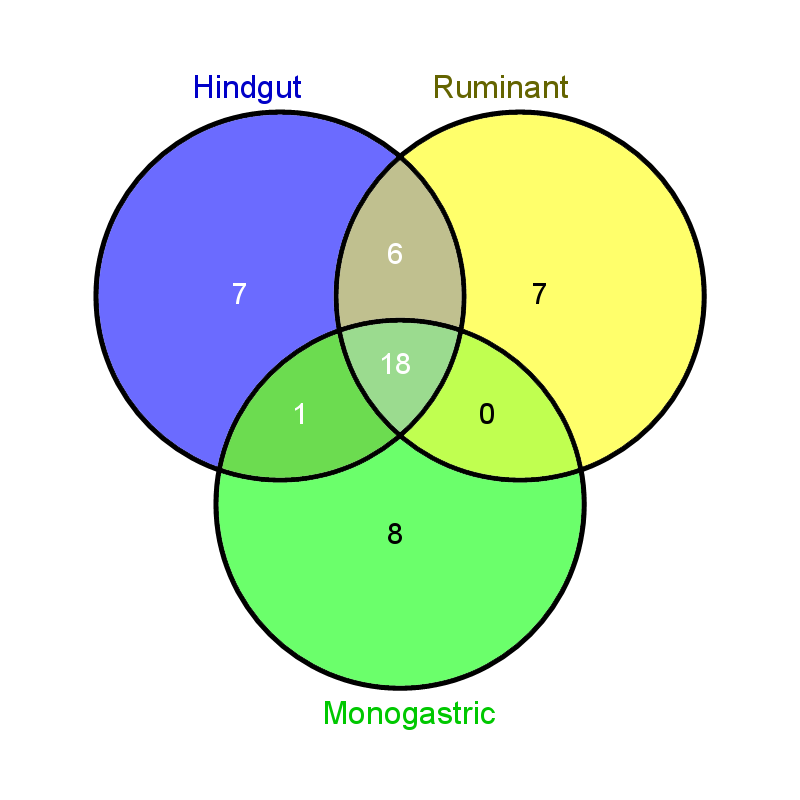
**

Figure S1. Venn diagram representation of the number of shared, core and unique genera in the microbiota of the ruminant, hindgut fermenters and mono-gastric animals.

**
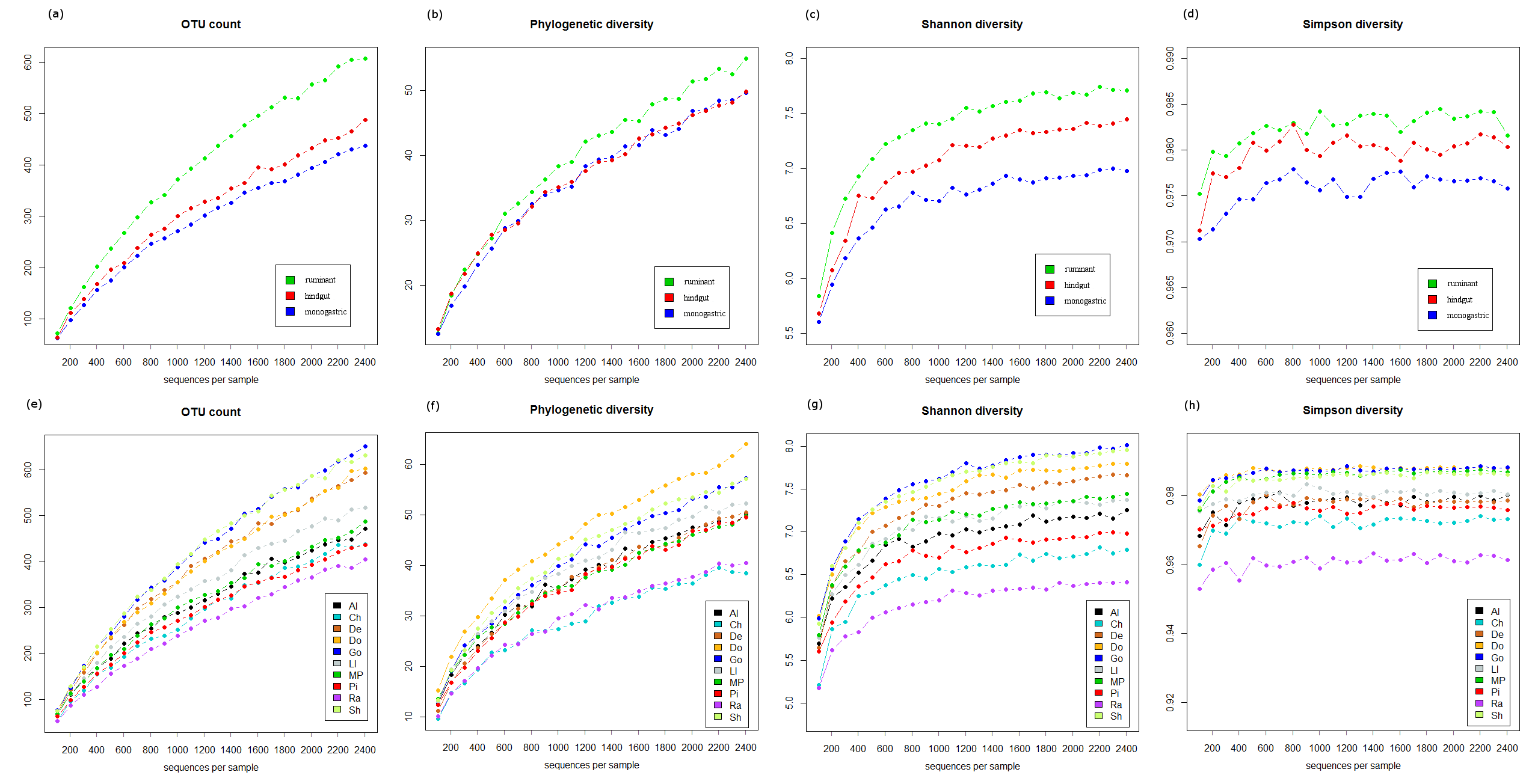
**

Figure S2. Alpha diversity rarefaction curves. (a) OTU count for each digestion type (b) Phylogenetic diversity indices for each digestion type (c) Shannon diversity indices for each digestion type (d) Simpson diversity indices for each digestion type (e) OTU count for each animal species (f) Phylogenetic diversity indices for each animal species (g) Shannon diversity indices for each animal species (h) Simpson diversity indices for each animal species.


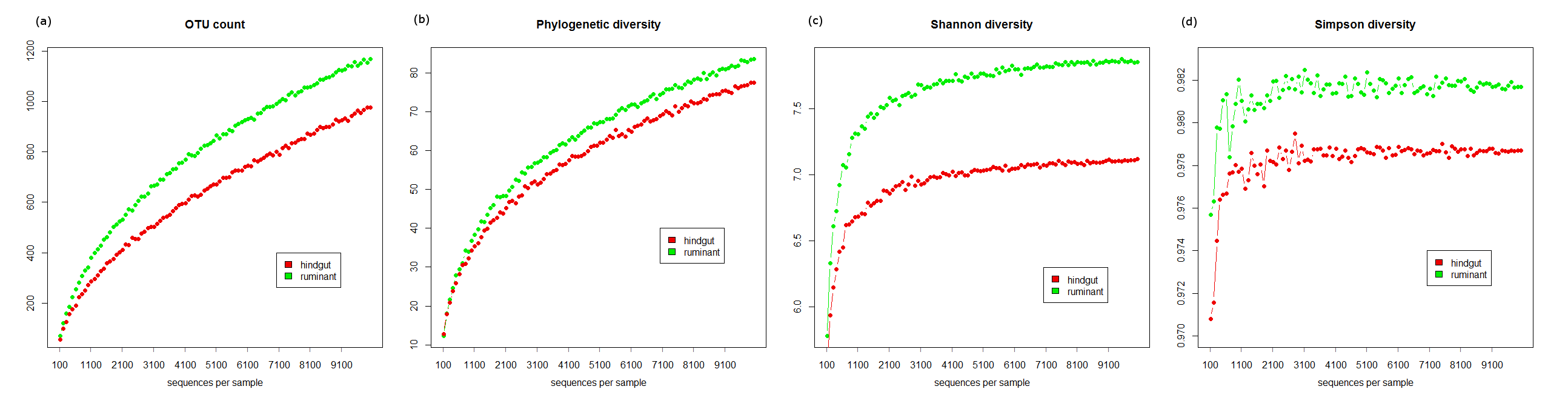


Figure S3. Rarefaction curves for two digestion types**;** hindgut fermenters and ruminants, using a 10,000 subset sample.

| **Table S1.** Classification of the animals studied | | | | | |
| --- | --- | --- | --- | --- | --- |
| Animal | Taxon level | | | | |
|  | Kingdom | Phylum | Class | Order | Family |
| Chinchilla | *Animalia* | *Chordata* | *Mammalia* | *Rodentia* | *Chinchillidae* |
| Rabbit | *Animalia* | *Chordata* | *Mammalia* | *Lagomorpha* | *Leporidae* |
| Donkey | *Animalia* | *Chordata* | *Mammalia* | *Perissodactyla* | *Equidae* |
| Miniature ponies | *Animalia* | *Chordata* | *Mammalia* |  |  |
| Deer | *Animalia* | *Chordata* | *Mammalia* | *Artiodactyla* | *Cervidae* |
| Goats | *Animalia* | *Chordata* | *Mammalia* |  | *Bovidae* |
| Sheep | *Animalia* | *Chordata* | *Mammalia* |  |  |
| Llama | *Animalia* | *Chordata* | *Mammalia* |  | *Camelidae* |
| Alpaca | *Animalia* | *Chordata* | *Mammalia* |  |  |
| Pigs | *Animalia* | *Chordata* | *Mammalia* |  | *Suidae* |

| Table S2. Statistically significant differences in the taxon abundance in the microbiota of hindgut fermenters and ruminants | | | |
| --- | --- | --- | --- |
| Taxa | Median read percentages (%) | | P-value |
|  | Hindgut | Ruminant |  |
| **Phylum** |  |  |  |
| *Firmicutes* | 53.111 | 65.346 | * |
| *Actinobacteria* | 0.176 | 0.018 | ** |
| **Class** |  |  |  |
| *Clostridia* | 45.912 | 62.651 | ** |
| *Bacilli* | 0.373 | 0.117 | * |
| *Gammaproteobacteria* | 0.336 | 0.056 | ** |
| *Alphaproteobacteria* | 0.235 | 0.451 | * |
| *Actinobacteria* | 0.176 | 0.018 | ** |
| *Deltaproteobacteria* | 0.175 | 0.372 | * |
| *Betaproteobacteria* | 0.133 | 0.013 | * |
| *Epsilonproteobacteria* | 0.018 | 0.000 | * |
| **Order** |  |  |  |
| *Clostridiales* | 44.088 | 60.725 | * |
| *Lactobacillales* | 0.306 | 0.065 | * |
| *Pseudomonadales* | 0.117 | 0.011 | * |
| *Burkholderiales* | 0.077 | 0.013 | * |
| *Desulfovibrionales* | 0.037 | 0.012 | * |
| *Aeromonadales* | 0.035 | 0.013 | * |
| *Bifidobacteriales* | 0.030 | 0.000 | * |
| *Campylobacterales* | 0.018 | 0.000 | * |
| *Rickettsiales* | 0.000 | 0.062 | * |
| **Family** |  |  |  |
| *Ruminococcaceae* | 20.480 | 33.461 | ** |
| *Marinilabiaceae* | 0.402 | 0.002 | ** |
| *Bacteroidaceae* | 0.363 | 1.853 | * |
| *Clostridiaceae* | 0.274 | 0.445 | * |
| *Lactobacillaceae* | 0.172 | 0.041 | ** |
| *Rikenellaceae* | 0.107 | 3.083 | *** |
| *Moraxellaceae* | 0.083 | 0.007 | * |
| *Incertae Sedis XIII* | 0.050 | 0.142 | ** |
| *Desulfovibrionaceae* | 0.030 | 0.007 | * |
| *Bifidobacteriaceae* | 0.030 | 0.000 | * |
| *Peptostreptococcaceae* | 0.000 | 0.104 | *** |
| *Gracilibacteraceae* | 0.000 | 0.312 | *** |
| *Rickettsiaceae* | 0.000 | 0.052 | * |
| **Genus** |  |  |  |
| *Faecalibacterium^a^* | 1.102 | 0.344 | ** |
| *Acetivibrio^a^* | 0.927 | 1.247 | ** |
| *Prevotella^a^* | 0.915 | 0.365 | * |
| *Oscillibacter^a^* | 0.709 | 1.554 | *** |
| *Papillibacter^a^* | 0.449 | 1.651 | *** |
| *Paludibacter* | 0.444 | 1.424 | * |
| *Coprococcus^a^* | 0.418 | 0.894 | *** |
| *Anaerophaga* | 0.402 | 0.002 | ** |
| *Bacteroides^a^* | 0.363 | 1.853 | * |
| *Acidaminococcus* | 0.329 | 0.104 | * |
| *Lactobacillus* | 0.172 | 0.041 | ** |
| *Subdoligranulum* | 0.134 | 0.015 | ** |
| *Alistipes* | 0.107 | 3.083 | *** |
| *Acinetobacter* | 0.083 | 0.007 | * |
| *Parasporobacterium* | 0.078 | 0.020 | * |
| *Catenibacterium* | 0.078 | 0.025 | * |
| *Anaerostipes* | 0.069 | 0.000 | *** |
| *Holdemania* | 0.050 | 0.198 | * |
| *Desulfovibrio* | 0.030 | 0.007 | * |
| *Acetitomaculum* | 0.023 | 0.104 | * |
| *Acetanaerobacterium* | 0.015 | 0.199 | ** |
| *Lutispora* | 0.000 | 0.286 | *** |
| *Orientia* | 0.000 | 0.052 | * |

a – genera present in the core microbiota both at animal level and digestion method level.

* = <0.05, ** = <0.01, *** = <0.001. Note: Some of the values listed in the table as zero have at least 50% of the values are equal to zero.
